# Medicaid expansion is associated with treatment receipt, timeliness, and outcomes among young adults with breast cancer

**DOI:** 10.1093/jncics/pkad067

**Published:** 2023-09-14

**Authors:** Xu Ji, Kewei Sylvia Shi, Kathryn J Ruddy, Jingxuan Zhao, Ann C Mertens, K Robin Yabroff, Sharon M Castellino, Xuesong Han

**Affiliations:** Department of Pediatrics, Emory University School of Medicine, Atlanta, GA, USA; Aflac Cancer & Blood Disorders Center, Children’s Healthcare of Atlanta, Atlanta, GA, USA; Surveillance and Health Equity Science, American Cancer Society, Atlanta, GA, USA; Department of Oncology, Mayo Clinic, Rochester, MN, USA; Surveillance and Health Equity Science, American Cancer Society, Atlanta, GA, USA; Department of Pediatrics, Emory University School of Medicine, Atlanta, GA, USA; Aflac Cancer & Blood Disorders Center, Children’s Healthcare of Atlanta, Atlanta, GA, USA; Department of Epidemiology, Emory Rollins School of Public Health, Atlanta, GA, USA; Surveillance and Health Equity Science, American Cancer Society, Atlanta, GA, USA; Department of Pediatrics, Emory University School of Medicine, Atlanta, GA, USA; Aflac Cancer & Blood Disorders Center, Children’s Healthcare of Atlanta, Atlanta, GA, USA; Surveillance and Health Equity Science, American Cancer Society, Atlanta, GA, USA

## Abstract

Female breast cancer is a common cancer in young adults, an age group with the highest uninsured rate. Among 51 675 young adult women (ages 18-39 years) diagnosed with breast cancer between 2011 and 2018 in the National Cancer Database, we estimated changes in guideline-concordant treatment receipt, treatment timeliness, and survival associated with the Affordable Care Act Medicaid expansion. Of young adults with stage I-III estrogen receptor–positive or progesterone receptor–positive breast cancer, Medicaid expansion was associated with a net increase of 2.42 percentage points (95% confidence interval [CI] = 0.56 to 4.28 percentage points) in the percentage receiving endocrine therapy. Among all young adults with stage I-III breast cancer, Medicaid expansion was associated with a net reduction of 1.65 percentage points (95% CI = 0.08 to 3.22 percentage points) in treatment delays defined as treatment initiation of at least 60 days after diagnosis and a net increase of 1.00 percentage points (95% CI = 0.21 to 1.79 percentage points) in 2-year overall survival. Our study provides evidence of benefit in cancer care and outcomes from Medicaid expansion among the young adult population.

Female breast cancer is among the most common young adult cancers (diagnosed at ages 18-39 years) (1), with approximately 10 850 young adult women diagnosed with invasive breast cancer in 2022 (2). Young adults with breast cancer experience more advanced stage diagnoses and worse prognosis than older patients (3), partially because of a lack of insurance coverage for timely access to guideline-concordant care (4). Our prior study has shown that Medicaid expansion under the Affordable Care Act (ACA) increases insurance coverage and early stage cancer diagnoses for young adults (5), but little research has examined changes in breast cancer treatment and downstream outcomes following Medicaid expansion. We examined the association of Medicaid expansion with receipt of timely, guideline-concordant treatment and survival among young adults with breast cancer.

The National Cancer Database (NCDB), a nationwide cancer registry, captures more than 80% of all new young adult cancer cases in the United States (6). Our sample comprised women diagnosed with a first primary, invasive breast cancer at ages 18-39 years during 2011-2018. We ensured follow-up of at least 12 months for all patients and excluded patients with missing information on diagnosis or treatment dates, stage, follow-up, self-reported race and ethnicity, or zip code–level income (Supplementary Figure 1, available online). This study was approved by the institutional review boards of the Morehouse School of Medicine and by Emory University under exempt review.

Guideline-concordant treatment receipt was measured as the receipt of 1) any endocrine therapy among young adults with estrogen receptor–positive or progesterone receptor–positive breast cancer; 2) any chemotherapy or targeted therapy among young adults with estrogen receptor–negative and progesterone receptor–negative breast cancer; and 3) the first appropriate treatment among all young adults, defined as surgery, chemotherapy, or targeted therapy for stage I-III diagnoses and systemic therapy for stage IV diagnoses (7). Notably, neoadjuvant endocrine therapy was considered as a first appropriate treatment for patients diagnosed with stage I-III estrogen receptor–positive breast cancer in 2016 or after, to be consistent with the 2016 National Comprehensive Cancer Network guideline update (8). Treatment timeliness was measured as initiating the first appropriate treatment less than 60 days from diagnosis. Sensitivity analysis of less than 90 days was conducted (9). We further assessed 2-year overall survival. Pre-expansion, young adults were followed from diagnosis until 3 months before states’ Medicaid expansion, last contact date, or death date, whichever occurred first. Postexpansion, young adults were followed from diagnosis until December 31, 2019, last contact, or death date, whichever came first.

We applied the difference-in-differences method to estimate changes in study outcomes post- vs pre-Medicaid expansion in expansion vs nonexpansion states (Supplementary Table 1, available online) (5,10,11). The difference-in-differences approach accounts for secular trend and common shocks (eg, the ACA Dependent Coverage Expansion provision) that may affect the study outcomes (12). Postexpansion starts on the date when expansion states implemented Medicaid expansion or on January 1, 2014, for nonexpansion states. We confirmed the pre-expansion parallel trend assumption of difference-in-differences models by restricting data to the pre-expansion period and interacting residence in expansion (vs nonexpansion) states with quarterly time trend (Supplementary Table 2, available online).

Linear probability models estimated dichotomous treatment outcomes. Flexible parametric survival models estimated survival (10,11). Difference-in-differences models adjusted for age, self-reported race and ethnicity, rurality, zip code–level income, comorbidity, and diagnosis year, with standard errors clustered at the state level. All models were stratified by diagnosis stage (stage I-III vs IV). Statistical significance was set at .05; all tests were 2-sided.

Among young adults with stage I-III estrogen receptor–positive or progesterone receptor–positive breast cancer (n = 33 166), the percentage receiving endocrine therapy increased in expansion states (85.21% pre-expansion to 86.46% postexpansion) but decreased in nonexpansion states (84.32% to 82.79%), leading to a net increase of 2.42 percentage points (95% confidence interval [CI] = 0.56 to 4.28 percentage points) in adjusted difference-in-differences models (Table 1;  Supplementary Table 3, available online). Changes in other treatment receipt measures for stage I-III patients were nonsignificant as were changes in all treatment receipt measures for stage IV patients.

**Table 1.** Changes in treatment receipt and timeliness following the implementation of Medicaid expansion among young adult women diagnosed with breast cancer, by cancer stage^a^

Measures of treatment receipt and timeliness	No.	Expansion states			Nonexpansion states			Unadjusted model		Adjusted model^b^	
		Pre–Medicaid expansion,^c^ %	Post–Medicaid expansion, ^c^ %	Percentage point absolute difference (95% CI)	Pre–Medicaid expansion ,^c^ %	Post–Medicaid expansion,^c^ %	Percentage point absolute difference (95% CI)	Percentage point difference-in-differences (95% CI)	*P*	Percentage point difference-in-differences (95% CI)	*P*
Among young adults with stage I-III											
Received any endocrine therapy (of those classified as estrogen receptor– or progesterone receptor–positive)	33 166	85.21	86.46	1.25 (0.12 to 2.39)	84.32	82.79	−1.52 (−3.00 to -0.05)	2.77 (0.91 to 4.64)	.003	2.42 (0.56 to 4.28)	.011
Received any chemotherapy or targeted therapy (of those classified as estrogen receptor– and progesterone receptor–negative)	13 062	95.14	95.00	−0.14 (−1.24 to 0.96)	94.73	94.88	0.15 (−1.25 to 1.55)	−0.29 (−2.07 to 1.49)	.749	−0.24 (−2.03 to 1.54)	.788
Received the first appropriate treatment (ie, surgery, chemotherapy, or targeted therapy)	48 360	99.11	99.02	−0.09 (−0.36 to 0.18)	98.94	98.64	−0.31 (−0.65 to 0.04)	0.22 (−0.22 to 0.66)	.336	0.22 (−0.22 to 0.67)	.318
Received the first appropriate treatment within 60 days from diagnosis	48 360	83.60	82.17	−1.43 (−2.41 to -0.45)	86.59	83.88	−2.72 (−3.96 to -1.48)	1.28 (−0.3 to 2.86)	.111	1.65 (0.08 to 3.22)	.040
Received the first appropriate treatment within 90 days from diagnosis	48 360	94.43	94.19	−0.24 (−0.85 to 0.37)	95.21	94.19	−1.03 (−1.8 to -0.26)	0.79 (−0.19 to 1.77)	.115	0.99 (0.01 to 1.97)	.047
Among young adults with stage IV											
Received any endocrine therapy (of those classified as estrogen receptor– or progesterone receptor–positive)	2297	70.48	73.39	2.91 (−2.64 to 8.45)	65.48	70.89	5.41 (−2.17 to 12.99)	−2.50 (−11.90 to 6.89)	.601	−2.37 (−11.73 to 6.98)	.619
Received any chemotherapy or targeted therapy (of those classified as estrogen receptor– and progesterone receptor–negative)	807	92.63	94.46	1.83 (−3.17 to 6.83)	95.00	96.12	1.12 (−4.97 to 7.2)	0.71 (−7.17 to 8.59)	.859	1.05 (−6.87 to 8.97)	.794
Received the first appropriate treatment (ie, systemic therapy)	3315	94.06	95.76	1.70 (−0.38 to 3.78)	96.09	96.48	0.39 (−2.35 to 3.13)	1.31 (−2.13 to 4.75)	.454	1.28 (−2.16 to 4.72)	.467
Received the first appropriate treatment within 60 days from diagnosis	3315	79.41	83.89	4.48 (0.60 to 8.36)	81.64	83.80	2.16 (−2.96 to 7.27)	2.32 (−4.09 to 8.74)	.478	2.51 (−3.89 to 8.91)	.441
Received the first appropriate treatment within 90 days from diagnosis	3315	89.17	92.04	2.87 (−0.04 to 5.78)	89.45	91.76	2.31 (−1.53 to 6.14)	0.56 (−4.25 to 5.38)	.818	0.59 (−4.22 to 5.4)	.810

a Authors’ analysis of the National Cancer Database. CI = confidence interval.

b Regression models also adjusted for age group, race and ethnicity, residence metropolitan statistical area status, zip code–level income, Charlson comorbidity score, and year of diagnosis. Standard errors were clustered at the state level.

c Pre- vs postexpansion status was defined based on the implementation date of Medicaid expansion through the Affordable Care Act. For the 24 states and Washington, DC, that expanded Medicaid in January 2014, and for nonexpansion states, postexpansion starts on January 1, 2014. For the 7 late-expansion states, postexpansion starts on the exact date after January 2014, when the state implemented Medicaid expansion. See more details in Supplementary Table 1 (available online).

Among young adults with stage I-III breast cancer (n = 48 360), the percentage initiating treatment less than 60 days after diagnosis decreased less in expansion (83.60% to 82.17%) than nonexpansion (86.59% to 83.88%) states, resulting in a net reduction of 1.65 percentage points (95% CI = 0.08 to 3.22 percentage points) in treatment delays in adjusted difference-in-differences models (Table 1). Similar patterns were observed for treatment initiation less than 90 days after diagnosis (adjusted difference-in-differences = 0.99 percentage points, 95% CI = 0.01 to 1.97 percentage points). Among stage IV patients (n = 3315), Medicaid expansion was positively associated with timely treatment initiation but statistically nonsignificant.

Among all young adults (n = 51 675), 2-year survival increased in expansion states (95.38% to 95.52%) but decreased in nonexpansion states (95.99% to 94.61%), contributing to a net survival increase of 1.46 percentage points (95% CI = 0.52 to 2.40 percentage points) in adjusted difference-in-differences models (Table 2). Similar patterns were seen in stage I-III patients (adjusted difference-in-differences = 1.00 percentage points, 95% CI = 0.21 to 1.79 percentage points). In stage IV patients, Medicaid expansion was positively associated with survival but statistically nonsignificant.

**Table 2.** Changes in 2-year overall survival following the implementation of Medicaid expansion among young adult women diagnosed with breast cancer, total and by cancer stage^a^

2-year overall survival^b^	No.	Expansion states			Nonexpansion states			Unadjusted model		Adjusted model^c^	
		Pre–Medicaid expansion,^d^ %	Post–Medicaid expansion,^d^ %	Percentage point absolute difference (95% CI)	Pre–Medicaid expansion,^d^ %	Post–Medicaid expansion,^d^ %	Percentage point absolute difference (95% CI)	Percentage point difference-in-differences (95% CI)	*P*	Percentage point difference-in-differences (95% CI)	*P*
Total	51 675	95.38	95.52	0.14 (−0.46 to 0.74)	95.99	94.61	−1.38 (−2.10 to −0.66)	1.52 (0.58 to 2.46)	.001	1.46 (0.52 to 2.40)	.002
Among young adults with stage I-III	48 360	96.84	96.99	0.15 (−0.37 to 0.68)	97.24	96.30	−0.93 (−1.57 to −0.29)	1.09 (0.26 to 1.92)	.010	1.00 (0.21 to 1.79)	.014
Among young adults with stage IV	3315	73.13	75.53	2.40 (−2.47 to 7.26)	70.87	68.31	−2.56 (−9.12 to 3.99)	4.96 (−3.19 to 13.11)	.233	4.55 (−3.51 to 12.60)	.269

a Authors’ analysis of the National Cancer Database. CI = confidence interval.

b Pre-expansion, young adults were followed from diagnosis until 3 months before their state’s Medicaid expansion, last contact date, or date of death, whichever occurred first. Postexpansion, young adults were followed from diagnosis until December 31, 2019, last contact, or death date, whichever came first. Notably, diagnoses postexpansion in Maine and Virginia were followed until September 30, 2018, as these 2 states, treated as nonexpansion states in this study, expanded Medicaid in January 2019. Moreover, diagnoses postexpansion in Utah and Idaho were followed until September 30, 2019, as these 2 states, treated as nonexpansion states in this study, expanded Medicaid in January 2020.

c Regression models also adjusted for age group, race and ethnicity, residence metropolitan statistical area status, zip code–level income, Charlson comorbidity score, and year of diagnosis. Standard errors were clustered at the state level.

d Pre- vs postexpansion status was defined based on the implementation date of Medicaid expansion through the Affordable Care Act. For the 24 states and Washington, DC, that expanded Medicaid in January 2014, and for nonexpansion states, postexpansion starts on January 1, 2014. For the 7 late-expansion states, postexpansion starts on the exact date after January 2014, when the state implemented Medicaid expansion. See more details in Supplementary Table 1 (available online).

Previous research has demonstrated associations of Medicaid expansion with increasing insurance coverage and early stage diagnoses among young adults with breast cancer (5). The young adult population is not mammographically screened, contributing to later stage at diagnosis and poorer prognosis. Our study adds evidence of increased receipt of timely, guideline-concordant treatment following Medicaid expansion among young adults with stage I-III breast cancer, suggesting that expansion-associated insurance gains may improve access to quality cancer care. Notably, the increased receipt of endocrine therapy in expansion states may reflect better adoption of the 2016 National Comprehensive Cancer Network guidelines update (recommending neoadjuvant endocrine therapy for early invasive estrogen receptor–rich breast cancer) in these states because of insurance coverage gains following the expansion (8).

However, we observed that stage I-III patients experienced a reduced likelihood of initiating the first appropriate treatment less than 60 days (or less than 90 days) in expansion and nonexpansion states. Such treatment delays may be a result of the increasing complexity of diagnostic process and treatment decisions (13). Importantly, our findings suggested that Medicaid expansion may reduce these delays. Improvements in access to care across the young adult breast cancer continuum following Medicaid expansion may explain the observed survival benefits. Notably, insurance gains through the expansion may improve access to retrieval therapy for relapse or progressive disease, follow-up care, and palliative care for young adults, an understudied area influencing overall survival and meriting future research.

This study has limitations. The NCDB is not population based; however, patient characteristics in the NCDB are comparable with population-based cancer registries (14,15). The small number of stage IV breast cancer patients may limit detection of differences. The NCDB lacks data on specific cause of death, an area meriting further investigation with other data sources, although other data sources may have less detailed treatment information and state coverage. Notably, given the focus on 2-year survival, most of the deaths may be attributable to cancer (16). The 2-year follow-up period is relatively short; continued follow-up of this cohort to assess long-term survival is critical in future research.

Our data showed increased receipt of certain guideline-concordant treatment, reduced treatment delays, and improved survival among young adults with stage I-III breast cancer associated with ACA Medicaid expansion. These findings provide implications to the 10 states that have not adopted Medicaid expansion to date (17) and to interventions aimed at increasing access as a means to improve breast cancer outcomes, with attention to years of life saved.

## Supplementary Material

pkad067_Supplementary_Data

## Data Availability

The data underlying this article were provided by the American College of Surgeons and accessed at the American Cancer Society by permission. The data cannot be shared publicly per the Data User Agreement. The National Cancer Database Participant User Files are available through application to investigators associated with the Commission on Cancer accredited cancer programs (https://www.facs.org/quality-programs/cancer/ncdb/puf).

## References

[1] Miller KD , Fidler-Benaoudia M, Keegan TH, Hipp HS, Jemal A, Siegel RL. Cancer statistics for adolescents and young adults, 2020.CA Cancer J Clin. 2020;70(6):443-459. 32940362 10.3322/caac.21637

[2] Giaquinto AN , Sung H, Miller KD, et al Breast cancer statistics, 2022. CA Cancer J Clin. 2022;72(6):524-541. 36190501 10.3322/caac.21754

[3] Murphy BL , Day CN, Hoskin TL, Habermann EB, Boughey JC. Adolescents and young adults with breast cancer have more aggressive disease and treatment than patients in their forties. Ann Surg Oncol. 2019;26(12):3920-3930. 31376035 10.1245/s10434-019-07653-9

[4] Conway D. *Uninsured Rates Highest For Young Adults Aged 19 to 34*. Suitland, Suitland-Silver Hill, MD: US Census Bureau; 2020. https://www.census.gov/library/stories/2020/10/uninsured-rates-highest-for-young-adults-aged-19-to-34.html. Accessed March 1, 2023.

[5] Ji X , Castellino SM, Mertens AC, et al Association of Medicaid expansion with cancer stage and disparities in newly diagnosed young adults. J Natl Cancer Inst. 2021;113(12):1723-1732. 34021352 10.1093/jnci/djab105 PMC9989840

[6] Robbins AS , Lerro CC, Barr RD. Insurance status and distant‐stage disease at diagnosis among adolescent and young adult patients with cancer aged 15 to 39 years: National Cancer Data Base, 2004 through 2010. Cancer. 2014;120(8):1212-1219. 24474656 10.1002/cncr.28568

[7] National Comprehensive Cancer Network. NCCN Guidelines Version 4. Breast Cancer. Plymouth Meeting, PA: National Comprehensive Cancer Network; 2023. https://www.nccn.org/guidelines/guidelines-detail?category=1&id=1419. Accessed March 22, 2023.

[8] Gradishar W , Salerno KE. NCCN guidelines update: breast cancer. J Natl Compr Canc Netw. 2016;14(suppl 5):641-644. 27226503 10.6004/jnccn.2016.0181

[9] Han X , Zhao J, Ruddy KJ, Lin CC, Sineshaw HM, Jemal A. The impact of dependent coverage expansion under the Affordable Care Act on time to breast cancer treatment among young women. PLoS One. 2018;13(6):e0198771. 29897976 10.1371/journal.pone.0198771 PMC5999229

[10] Han X , Zhao J, Yabroff RR, Johnson CJ, Jemal A. Association between medicaid expansion under the affordable care act and survival among newly diagnosed cancer patients. J Natl Cancer Inst. 2022;114(8):1176-1185. 35583373 10.1093/jnci/djac077 PMC9360456

[11] Ji X , Shi KS, Mertens AC, et al Survival in young adults with cancer is associated with Medicaid expansion through the affordable care act. J Clin Oncol. 2023;41(10):1909-1920. 36525612 10.1200/JCO.22.01742 PMC10082236

[12] Wooldridge JM. Econometric Analysis of Cross Section and Panel Data. Cambridge, MA: MIT press; 2010.

[13] Smith‐Graziani D , Lei X, Giordano SH, Zhao H, Karuturi M, Chavez‐MacGregor M. Delayed initiation of adjuvant chemotherapy in older women with breast cancer. Cancer Med. 2020;9(19):6961-6971. 32767723 10.1002/cam4.3363 PMC7541132

[14] Mallin K , Browner A, Palis B, et al Incident cases captured in the National Cancer Database compared with those in US population based central cancer registries in 2012–2014. Ann Surg Oncol. 2019;26(6):1604-1612. 30737668 10.1245/s10434-019-07213-1

[15] Surveillance Epidemiology and End Results (SEER) Program SEERStat Database. Incidence - SEER 18 Regs Research Data + Hurricane Katrina Impacted Louisiana Cases, Nov 2018 Sub (1975-2016 varying) - Linked To County Attributes - Total U.S., 1969-2017 Counties, National Cancer Institute, DCCPS, Surveillance Research Program. http://www.seer.cancer.gov. Released April 2019, based on the November 2018 submission.

[16] Afifi AM , Saad AM, Al‐Husseini MJ, Elmehrath AO, Northfelt DW, Sonbol MB. Causes of death after breast cancer diagnosis: a US population‐based analysis. Cancer. 2020;126(7):1559-1567. 31840240 10.1002/cncr.32648

[17] Kaiser Family Foundation. Status of State Medicaid Expansion Decisions: Interactive Map. Menlo Park, CA*:* Kaiser Family Foundation; 2023. https://www.kff.org/medicaid/issue-brief/status-of-state-medicaid-expansion-decisions-interactive-map/. Accessed December 15, 2022.

