# Supplementary material for: Medicaid expansion is associated with treatment receipt, timeliness, and outcomes among young adults with breast cancer
Source: JNCI Cancer Spectr. 2023 Sep 14;7(5):pkad067. doi: 10.1093/jncics/pkad067 (PMC10534051; doi:10.1093/jncics/pkad067)

Supplementary Material

Supplementary Table 1: State Medicaid expansion status between 2011 and 2018

Expansion states:	
24 states and D.C. that expanded Medicaid in January 1, 2014	California, Connecticut, Minnesota, New Jersey, Washington, Arizona, Arkansas, Colorado, Delaware, Hawaii, Illinois, Iowa, Kentucky, Maryland, Massachusetts, Nevada, New Mexico, New York, North Dakota, Ohio, Oregon, Rhode Island, Vermont, West Virginia, D.C.
7 late-expansion states	Michigan [April 2014], New Hampshire [August 2014], Pennsylvania [January 2015], Indiana [February 2015], Alaska [September 2015], Montana [January 2016], Louisiana [July 2016])
Non-expansion states:	
19 non-expansion states (which did not implement Medicaid expansion during 2011-2018)	Alabama, Florida, Georgia, Idaho, Kansas, Maine, Mississippi, Missouri, Nebraska, North Carolina, Oklahoma, South Carolina, South Dakota, Tennessee, Texas, Utah, Virginia, Wisconsin, Wyoming

Supplementary Table 2: Results from the test for the "pre-policy parallel trend" assumption of the difference-in-differences method

Outcomes	P-value for expansion-by-time interaction term	
	Unadjusted model [a]	Adjusted model [a,b]
2-year overall survival		
Total	0.282	0.321
Among YAs with stage I-III	0.127	0.120
Among YAs with stage IV	0.650	0.437
Treatment outcomes among YAs with stage I-III		
Received any endocrine therapy (of those classified as ER+ or PR+)	0.001	0.003
Received any chemotherapy or targeted therapy (of those classified as ER- and PR-)	0.706	0.722
Received the first appropriate treatment (i.e., surgery, chemo, or targeted therapy)	0.341	0.348
Received the first appropriate treatment within 60 days from diagnosis	0.149	0.125
Received the first appropriate treatment within 90 days from diagnosis	0.270	0.245
Treatment outcomes among YAs with stage IV		
Received any endocrine therapy (of those classified as ER+ or PR+)	0.766	0.833
Received any chemotherapy or targeted therapy (of those classified as ER- and PR-)	0.831	0.946
Received the first appropriate treatment (i.e., systemic therapy)	0.973	0.960
Received the first appropriate treatment within 60 days from diagnosis	0.143	0.160
Received the first appropriate treatment within 90 days from diagnosis	0.689	0.745

Notes: Authors' analysis of the National Cancer Database.
YA: young adults. ER: Estrogen Receptor. PR: Progesterone Receptor.
[a] The "Pre-expansion Parallel Trend" assumption of the difference-in-differences models was tested by restricting our sample to pre-expansion diagnoses and grouping the pre-expansion diagnoses into quarter intervals. We then estimated the regression model used in our main analysis by including an expansion-by-quarter interaction term, for each study outcome. The p-value of the expansion-by-quarter interaction term in these regression models was reported in this table.
[b] These models also adjusted for age group, race and ethnicity, zip code-level income, residence metropolitan statistical area status, Charlson comorbidity score, and year of diagnosis, with standard errors clustered at the state level.

Supplementary Table 3: Characteristics of YA women newly diagnosed with breast cancer (aged 18-39 years at diagnosis), using the 2011-2018 National Cancer Database

Characteristics	Total<br>N=51675	Among YAs with Stage I-III<br>N=48360	Among YAs with Stage IV<br>N=3315
Medicaid expansion states			
Expansion states	31217 (60.4)	29238 (60.5)	1979 (59.7)
Non-expansion states	20458 (39.6)	19122 (39.5)	1336 (40.3)
Year of cancer diagnosis			
2011	6661 (12.9)	6263 (13.0)	398 (12.0)
2012	5448 (10.5)	5155 (10.7)	293 (8.8)
2013	491 (1.0)	469 (1.0)	22 (0.7)
2014	6785 (13.1)	6381 (13.2)	404 (12.2)
2015	7612 (14.7)	7120 (14.7)	492 (14.8)
2016	8129 (15.7)	7564 (15.6)	565 (17.0)
2017	8190 (15.9)	7631 (15.8)	559 (16.9)
2018	8359 (16.2)	7777 (16.1)	582 (17.6)
Age at cancer diagnosis (years)			
18-25	1423 (2.8)	1290 (2.7)	133 (4.0)
26-34	19842 (38.4)	18438 (38.1)	1404 (42.4)
35-39	30410 (58.9)	28632 (59.2)	1778 (53.6)
Race and ethnicity			
Non-Hispanic White	32724 (63.3)	30832 (63.8)	1892 (57.1)
Race/ethnic minority groups combined[§]	18951 (36.7)	17528 (36.2)	1423 (42.9)
Zip code-level median household income			
Low (≤138 FPL)	3642 (7.1)	3272 (6.8)	370 (11.2)
Middle (139-400 FPL)	42841 (82.9)	40123 (83.0)	2718 (82.0)
High (>401 FPL)	5192 (10.1)	4965 (10.3)	227 (6.9)
Residence metropolitan statistical area status			
Metropolitan	44912 (86.9)	42070 (87.0)	2842 (85.7)
Non-Metropolitan	5091 (9.9)	4749 (9.8)	342 (10.3)
Unknown	1672 (3.2)	1541 (3.2)	131 (4.0)
Charlson comorbidity score			
0	49938 (96.6)	46732 (96.6)	3206 (96.7)
≥1	1737 (3.4)	1628 (3.4)	109 (3.3)
Breast cancer subtype[⁋]			
ER+ or PR+	35463 (68.6)	33166 (68.6)	2297 (69.3)
ER- and PR-	13869 (26.9)	13062 (27.0)	807 (24.4)
Unknown subtype	2343 (4.5)	2132 (4.4)	211 (6.4)

Notes: Authors' analysis of the National Cancer Database.
YA: young adult. FPL: federal poverty level. ER: Estrogen Receptor. PR: Progesterone Receptor.
[§] Included Hispanic, non-Hispanic Black, non-Hispanic others (including Asian, American Indian or Alaska Native, and Native Hawaiian or other Pacific Islander), and unknown race/ethnicity.
[⁋] The receptor status was categorized based on risk stratification. ER was coded using SSF1 (Estrogen Receptor Assay), and PR was coded using SSF2 (Progesterone Receptor Assay), according to the Surveillance, Epidemiology, and End Results (SEER) Registrar Staging Assistant (https://staging.seer.cancer.gov/cs/schema/02.05.50/breast/?breadcrumbs=(~schema_list~)%22).

Supplementary Figure 1: Analytic sample derivation flowchart

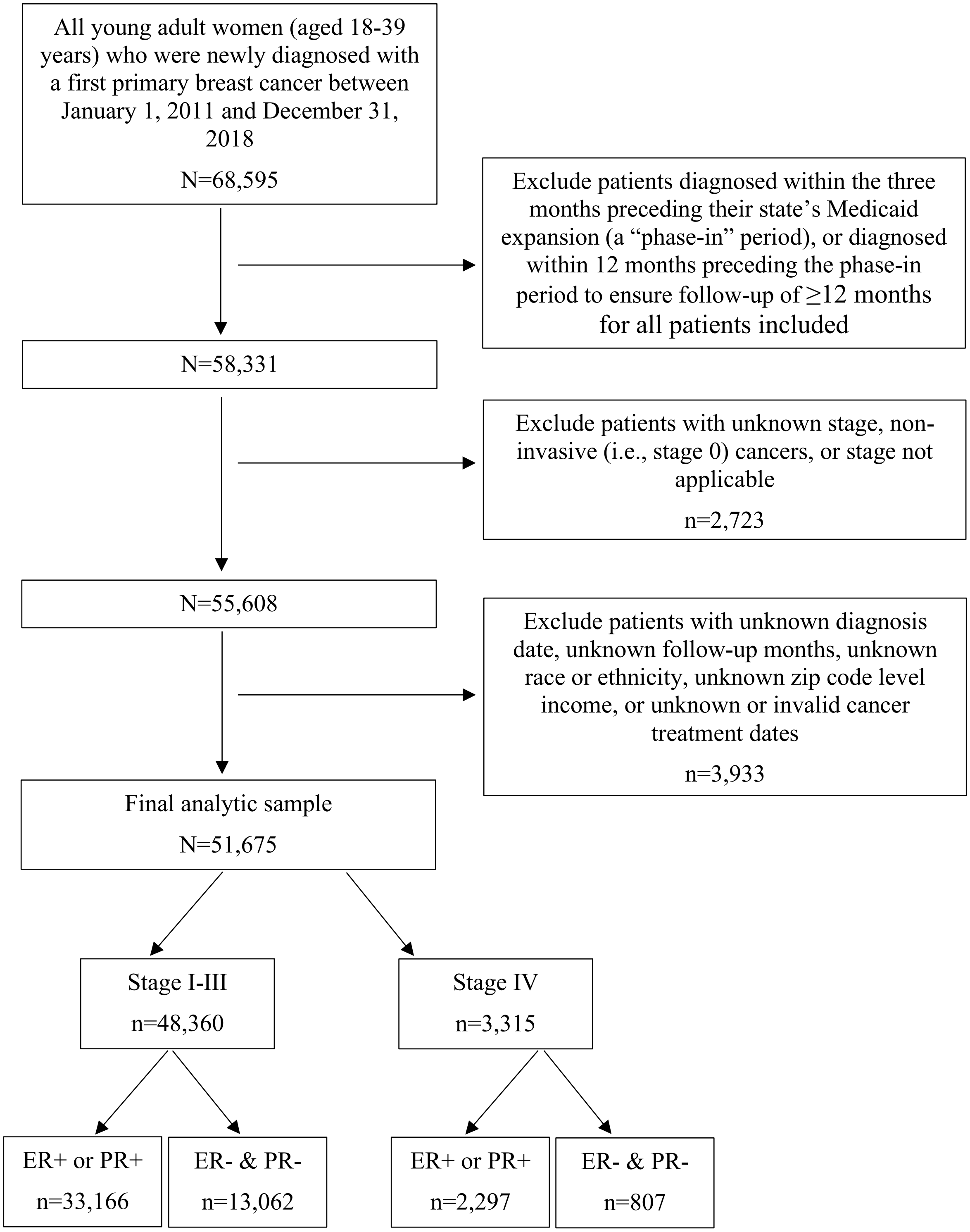

Supplement: pkad067_Supplementary_Data [file pkad067_supplementary_data.pdf]
